# The DECIDE project: from monitoring and surveillance data to decision-support for farmers and veterinarians

**DOI:** 10.12688/openreseurope.15988.2

**Published:** 2025-08-26

**Authors:** Gerdien van Schaik, Miel Hostens, Céline Faverjon, Dan B. Jensen, Anders R. Kristensen, Pauline Ezanno, Jenny Frössling, Fernanda Dórea, Britt-Bang Jensen, Luis Pedro Carmo, Wilma Steeneveld, Jonathan Rushton, William Gilbert, Angela Bearth, Michael Siegrist, Jasmeet Kaler, Johannes Ripperger, Jamuna Siehler, Sjaak de Wit, Beatriz Garcia-Morante, Joaquim Segalés, Bart Pardon, Jade Bokma, Mirjam Nielen

**Affiliations:** 1 Department of Population Health Sciences, Universiteit Utrecht, Utrecht, 3508TD, The Netherlands; 2 Royal GD, Deventer, The Netherlands; 3 Laboratory for Animal Nutrition and Animal Product Quality (Lanupro), Department of Animal Sciences and Aquatic Ecology, Ghent University, Gent, Belgium; 4 AUSVET Europe, Lyon, France; 5 Department of Veterinary and Animal Sciences, University of Copenhagen, Copenhagen, Denmark; 6 Oniris, INRAE, BIOEPAR, Nantes, France; 7 Department of Disease Control and Epidemiology, National Veterinary Institute, Uppsala, Sweden; 8 Section for Epidemiology, Norwegian Veterinary Institute, Oslo, Norway; 9 Institute of Infection and Global Health, University of Liverpool, Liverpool, England, UK; 10 Department of Health Sciences and Technology, Eidgenossische Technische Hochschule Zurich, Zürich, Zurich, Switzerland; 11 School of Veterinary Medicine and Science, University of Nottingham, Nottingham, England, UK; 12 accelopment Schweiz AG, Zurich, Switzerland; 13 Unitat Mixta d'Investigació IRTA-UAB en Sanitat Animal, Centre de Recerca en Sanitat Animal (CReSA), Universitat Autonoma de Barcelona, Barcelona, Catalonia, Spain; 14 IRTA Programes de Sanitat i Benestar Animals, Centre de Recerca en Sanitat Animal (CReSA), Universitat Autonoma de Barcelona, Barcelona, Catalonia, Spain; 15 Departament de Sanitat i Anatomia Animals, Facultat de Veterinària, UAB, Universitat Autonoma de Barcelona, Barcelona, Catalonia, Spain; 16 OIE Collaborating Centre for the Research and Control of Emerging and Re-Emerging Swine Diseases in Europe (IRTA-CReSA), Barcelona, Spain; 17 Department of Internal Medicine, Reproduction and Population Medicine, Ghent University, Gent, Belgium

**Keywords:** Data-driven, decision-support, control, endemic diseases, animal health, welfare

## Abstract

Farmers, veterinarians and other animal health managers in the livestock sector are currently missing sufficient information on the prevalence and burden of contagious endemic animal diseases. They need adequate tools for risk assessment and prioritization of control measures for these diseases. The DECIDE project develops data-driven decision-support tools, which present (i) robust and early signals of disease emergence and options for diagnostic confirmation; and (ii) options for controlling the disease along with their implications in terms of disease spread, economic burden and animal welfare. DECIDE focuses on respiratory and gastro-intestinal syndromes in the three most important terrestrial livestock species (pigs, poultry, cattle) and on reduced growth and mortality in two of the most important aquaculture species (salmon and trout). For each of these, we (i) identify the stakeholder needs; (ii) determine the burden of disease and costs of control measures; (iii) develop data sharing frameworks based on federated data access and meta-information sharing; (iv) build multivariate and multi-level models for creating early warning systems; and (v) rank interventions based on multiple criteria. Together, all of this forms decision-support tools to be integrated in existing farm management systems wherever possible and to be evaluated in several pilot implementations in farms across Europe. The results of DECIDE lead to improved use of surveillance data and evidence-based decisions on disease control. Improved disease control is essential for a sustainable food chain in Europe with increased animal health and welfare and that protects human health.

## Disclaimer

The views expressed in this article are those of the author(s). Publication in Open Research Europe does not imply endorsement of the European Commission.

## Introduction

Animal production is expected to intensify and expand due to an increasing demand for animal-derived food and the mounting pressure on land. Contagious livestock diseases impede the efficiency of animal production and lead to economic costs, poor animal welfare, and for certain diseases, have an impact on trade, public health, and consumer confidence. The European Union has a regulatory framework for all epizootic and other important contagious diseases listed in the Animal Health Regulation ( Regulation (EU) 2016/429). The situation of these regulated diseases is generally well known. However, the situation for endemic contagious diseases that the private sector is supposed to deal with is different. Farmers, veterinarians and other animal health managers in the livestock and aquaculture sectors are currently missing sufficient information on the prevalence and burden of contagious endemic animal diseases. Diseases, such as porcine reproductive and respiratory syndrome (PRRS) virus, avian infectious bronchitis, cardiomyopathy syndrome in salmon or bovine coronavirus infection, are estimated to cause 10–15% reduction in performance efficiency of livestock farming, resulting in large financial losses and lower sustainability as well as affecting animal welfare ( Delabouglise *et al.*, 2017) ( Mohd Nor *et al.*, 2012) ( Raasch *et al.*, 2020) ( Rushton *et al.*, 2018) ( Velarde *et al.*, 2015). Professionals in the livestock and aquaculture sectors are therefore in considerable need of adequate tools to assess the risks for contagion and associated losses, and to help prioritize the appropriate control measures for these diseases.

Existing on-farm data are numerous and heterogeneous, greatly limiting their direct use by farms, while they could be used to monitor deviations from the normal production processes indicative for infectious disease spread. ( Baldin *et al.,* 2021) conclude that the scientific community has not been successful in developing, disseminating, and promoting a sustained adoption of integrated decision support systems based on continuous, multiple data streams on dairy farms. While studies also show potential for data usage in disease surveillance and decision support systems ( Hepworth *et al.*, 2012; Niloofar *et al.,* 2021). Also, sensor data or (video) imaging, are currently already available in specific farms and species ( Halachmi *et al.*, 2019). For salmon production, imaging is already used quite extensively, while in terrestrial species, it is still in an early adoption phase. Salmon can thus serve as an example for the other species. Still, much progress could be made in managing endemic diseases by using available data in decision support systems useful to the farmers and their veterinarians ( Kasab-Bachi *et al.,* 2017; Rutten *et al.,* 2013) ( Van Limbergen *et al.,* 2020).

In this context, in 2020 the European Commission launched a call for projects to improve knowledge on endemic contagious diseases in livestock and aquaculture [ SFS-10-2020: Epidemiology of contagious animal diseases: from integrated data collection to prioritisation. ]. In response to the call, the project “Data-driven control and prioritisation of non-EU-regulated contagious animal diseases” ( DECIDE) was developed.

The DECIDE project develops data-driven decision-support tools that offer (i) robust and early signals of disease emergence and options for diagnostic confirmation, and (ii) options for controlling the disease along with their implications in terms of disease spread, economic burden and animal welfare. Together, all of this forms decision-support tools to be integrated in existing farm management systems wherever possible and to be evaluated in several pilot implementations in swine, poultry, cattle, and salmonid farms across Europe. The project started in 2021 and will reach conclusions in 2026.

The aim of this paper is to discuss the rationale behind the project and how the project tackles the challenges to eventually improve decisions to increase animal health and welfare.

## The concept of the DECIDE project

The main goal of DECIDE is to develop and evaluate data-driven decision-support tools that allow stakeholders in animal health and welfare management to make improved decisions on controlling endemic infectious animal diseases. DECIDE focuses on respiratory and gastro-intestinal syndromes in the three most important terrestrial livestock species (swine, poultry, and cattle) and on growth reduction and mortality in salmonids, the most important aquaculture species (salmon and trout).

**Figure 1.**
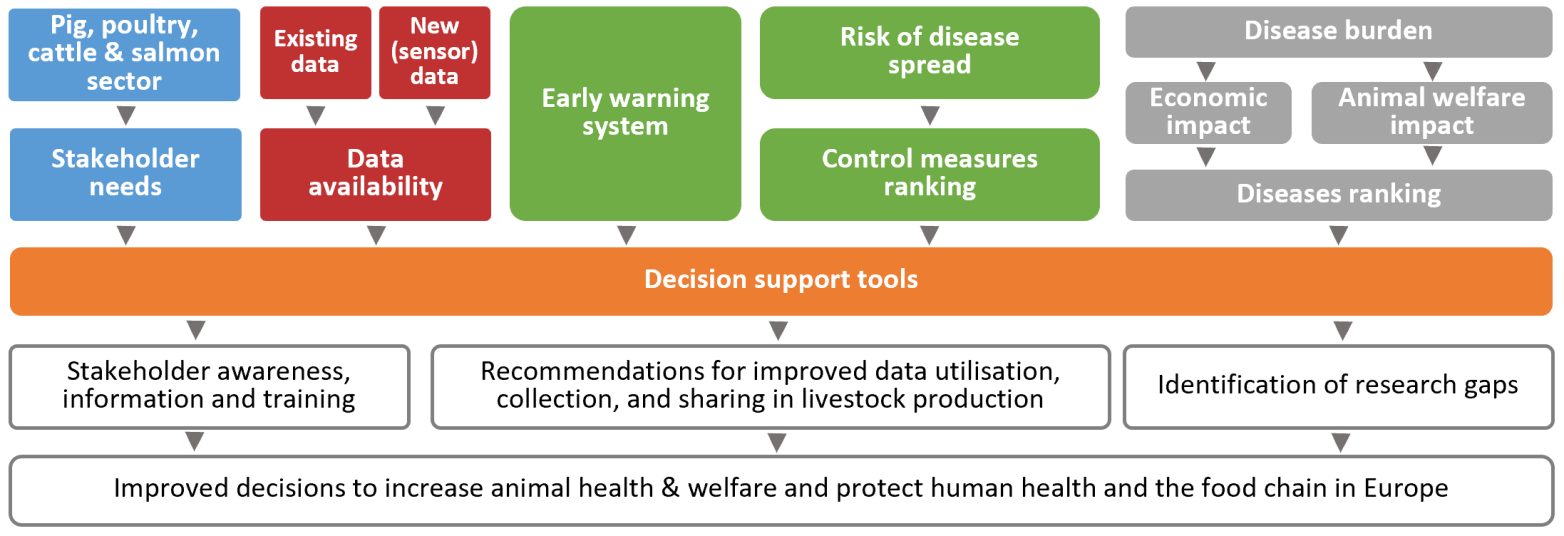
The concept of the DECIDE project.

The key ‘ingredients’ for such decision-support tools are the following ( Figure 1): (i) a clear view on the stakeholder needs, barriers and drivers; (ii) availability of and access to relevant data; (iii) early warning systems that can detect signals of potential disease emergence or spread; (iv) a set of potential control measures, along with associated costs; and (v) an understanding of the disease burden in terms of economic and welfare impact. The decision-support tool for a specific syndrome in a specific species gives various options. The tool ranks the possible control measures, including treatments and preventive measures for reduction of spread, economic costs and benefits and welfare implications. Based on embedded simulation models in the tool, stakeholders can see ‘what-if’ scenarios for a decision.

The data useful for DECIDE is existing data that can be used to monitor deviations from the normal production process indicative of infectious diseases spread. We also investigate the usefulness of novel data *e.g.* sensor data or (video) imaging. Data availability is crucial for the DECIDE project, but access of data is restricted by laws, regulations, privacy concerns, and market pressures, as individual actors operate in a competitive environment. To overcome these challenges and assure availability of and access to relevant data, procedures to ensure access to data from the different species are developed and documented. In addition, innovative approaches for supporting data access are investigated *e.g.*, federated access and federated learning ( Papst *et al.*, 2019).

The early warning system consists of data analysis that can detect aberrations from the normal variation in the monitoring data. “Early” should be interpreted in the sense that it detects signals indicative for disease timely enough for the stakeholders ( *e.g.* farmers, veterinarians) to be useful for decision making on disease-mitigating actions. Frequently updated visualisation of data helps the users understand and be aware of trends, including an immediate shift or deviation. The data information also confirms what the user already suspects and can be used to support general control strategies. The risk of disease spread is a key determinant for disease mitigating actions of the stakeholders. The effectiveness of control measures greatly depends on the ability to reduce further spread. Therefore, in DECIDE, mechanistic disease models are developed to simulate the spread of contagious diseases in the production systems of the four studied animal species. The input parameters of the models can easily be adapted to simulate spread of different pathogens, for different species and herd-types.

Stakeholders often have a choice of control measures including doing nothing at all. To effectively support stakeholders in their choice, a ranking of control measures for effectiveness, costs and benefits, welfare implications, medicine use, *etc.* is needed. Obviously, whether to control a disease or not also depends on the impact of that disease on animal health and welfare. DECIDE develops a multidimensional burden of disease metric that contains the economic impact as well as the impact on animal welfare and possibly on antimicrobial use. Thus, diseases can be ranked for their expected burden to prioritise the limited resources of the stakeholders for disease control.

The above-mentioned key ingredients are combined in decision-support tools for the stakeholders. The adoption of tools also require in-depth understanding of stakeholder beliefs, needs and preferences. Incorporating social science methods, the tools are developed in close collaboration with the stakeholder end-users. The decision-support tools are incorporated in already existing platforms to facilitate uptake of the tools. We develop prototypes for each species that are tested in several partner countries in pilot implementations. The gained understanding of the stakeholders’ needs and preferences does not only inform the development of the functionalities of these prototypes ( *i.e.*, what it can do), but also the output visualisation ( *i.e.*, how data is shown or how early warnings are framed). The pilot implementations are the ‘playground’ in which our innovative framework and tools are tested and adapted again, learning from experiences with stakeholders and users across species. Through the pilot implementations, we ensure that the generic methods and framework can be easily adapted and implemented for existing platforms. The long-term sustainability of the developed tools is ensured by an open science approach.

## The overall structure of the workplan

**Figure 2.**
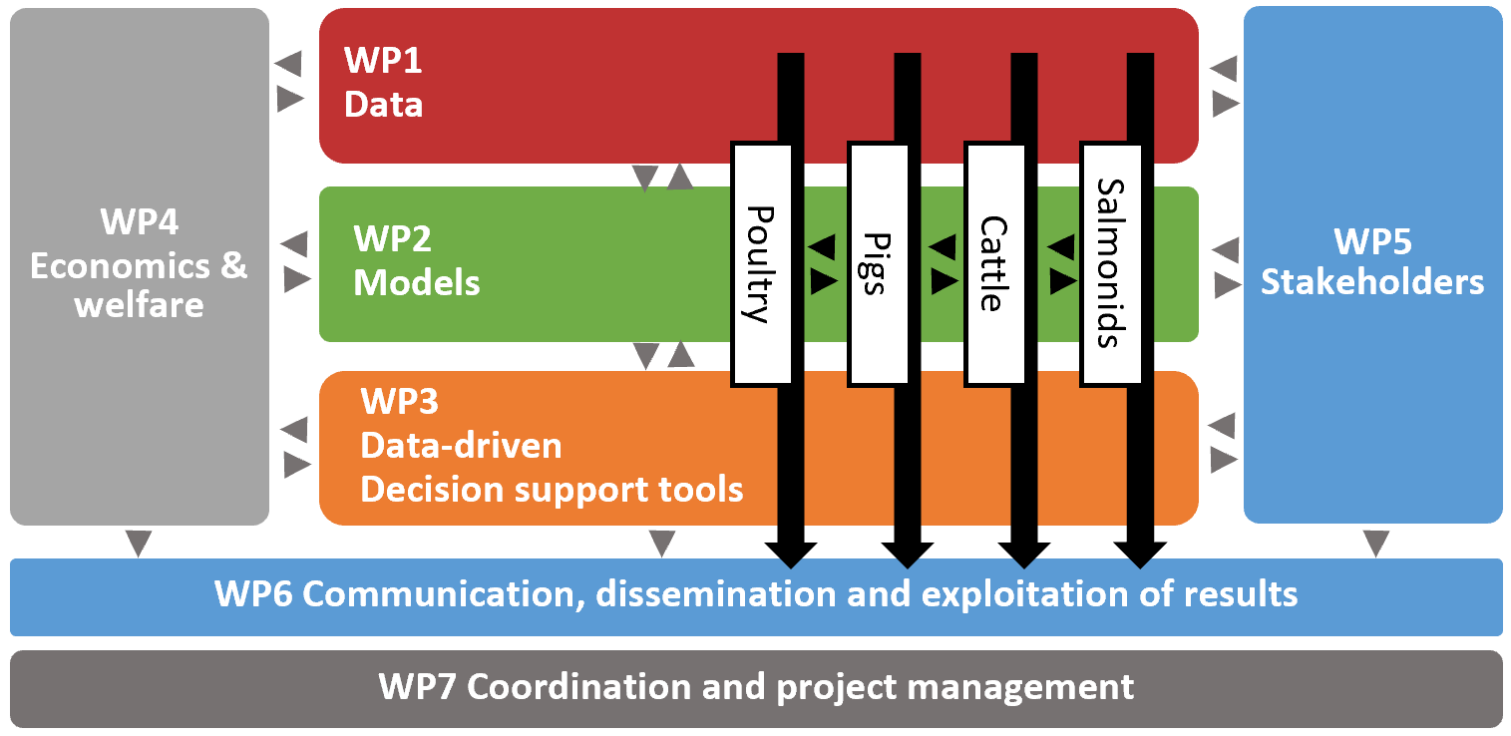
The work packages (WP) and species-specific workflows in the DECIDE project. Black arrows indicate the progress of the different species-specific decision-support tools through the WPs.

In Figure 2 the overall structure of the workplan of the DECIDE project is depicted. The DECIDE project consists of seven work packages (WP). WP1-WP3 cover all data-oriented research and tool development. WP4 is dedicated to determining the economic and welfare burden of diseases and to generate cost-effective control measures. For stakeholder input, WP5 aims to assess the stakeholders’ drivers, barriers and willingness to share data and to implement decision tools for health and welfare management at the farm level. The dissemination, communication and exploitation of results is organized in WP6, and the management and coordination of the project is overseen by WP7.

The technical work packages (WP1-5) in DECIDE are providing the necessary technical knowledge and scientific methods for the decision-support tools that are developed for each of the species in specific pilot implementations. The project is planned for a total duration of 5 years to give sufficient time for developing all ‘ingredients’ for the decision-support tools (in WP1, WP2, WP4 and WP5) and then for integrating and testing prototypes (WP3/WP5) of the decision-support tools in pilot implementations in at least two iterations within years 3 to 5. Some of the tools set up in WP3 may just be decision trees, while others are envisioned to be fully functional interactive dashboards. A key element of DECIDE is to build prototypes of the decision-support tools, one for each of the four species (pigs, poultry, cattle, salmonids). A prototype consists of generic models with country- and herd-specific baselines ( *e.g.*, (ab)normal mortality levels differ between herds and countries) to detect aberrations in the data for the different syndromes. Each of the prototypes is tested and further refined in pilot implementations in at least two different countries. The administrative workpackages WP6 and WP7 are for communication and dissemination (WP6) and overall project coordination (WP7).

## Content of the work packages

The main objective of WP1, Data, is to assure availability of and access to relevant data during the DECIDE project and to explore innovative approaches to combine, compare, and access data coming from numerous data sources for continuous use after DECIDE. As a first task in WP1 we review and compare data available in DECIDE, putting it in perspective with the General Data Protection Regulation (GDPR), and existing guidelines on best practices in usage of agricultural data, such as the ‘EU code of conduct on agricultural data sharing by contractual agreement and the ‘Privacy and security principles for farm data’ of the US Farm Bureau. Following the FAIR principles, machine-readable ontologies describing concepts within animal health are created for each of the animal domains, and shared definitions are built where possible ( Dórea *et al.*, 2019). Finally, different approaches to support data accessibility are tested and compared using as a basis the pilot implementations of the decision-support tool. A recent review ( Papst *et al.*, 2019) provides an overview of the existing agricultural data exchange systems and proposed options for privacy-preserving data analytics. Essentially, three alternative data exchange approaches are implemented and compared in WP1, namely (i) direct data sharing; (ii) centralized data exchange using federated data access; and (iii) privacy-preserving data analytics using federated learning.

The overall objective of WP2, Models, is to develop the methods for data analysis and modelling. In essence, two kinds of models are developed: i) monitoring models and ii) mechanistic simulation models. The purpose of a monitoring model is to use observed data to estimate the state of a system, which can be a group of animals, a herd, a region, or a country. The state can either be expressed quantitatively ( *e.g.* mortality or the prevalence of a syndrome) or qualitatively ( *e.g.* “in control” or “out of control” where “out of control” should raise an alarm). The observation is based on data, typically originating from multiple sources (sensors as well as manually collected). In many cases, the state is not directly observable. In other words, the data only indirectly reflect the state. Since the data from a biological system shows completely random fluctuations, monitoring the state also has a data filtering purpose. If the state is expressed quantitatively, the output from the monitoring model may serve as valuable input to a mechanistic simulation model of the system as well as for economics and welfare modelling (tackled in WP4). If the state is expressed qualitatively, the output may serve as valuable input to decision-support (focus of WP3). The monitoring framework consists of statistical models for time-series data that can detect aberrations in the data. Specifically, we use state space models, where a latent vector of parameters describes the state of the system ( Jensen *et al.*, 2017). Biologically meaningful disease-specific mechanistic models enable simulating pathogens’ spread and syndrome occurrence at different levels (pen, farm, region), and thus to compare *ex-ante* and prioritize control options ( Ezanno *et al.*, 2020). In DECIDE, the software EMULSION v1.1.1 ( Picault *et al.*, 2019) is used to facilitate the development of the stochastic simulation models.

In WP3, Data-driven decision-support tools, we focus on the development and implementation of tools to deliver information in formats that are usable (user-friendly) and useful (capable of providing insight to guide intervention and practice change) in supporting disease prioritization and control in practice. Tool prototypes are developed to the point that they can be used and tested, and evaluation is carried out in conjunction with WP2 and WP5 to further adapt to users’ needs for baseline models in a co-construction approach. Data visualization and information delivery tools vary from decision trees to fully functional interactive dashboards. After one or two rounds of feedback from stakeholders through participatory workshops, prototype support tools are built for personal computers, as well as smart phones and tablets, and tested in practice through the pilot implementations. These prototypes are applied in practice and used as a basis for discussions on users’ needs and evaluations of the tools’ usefulness and usability through the pilot implementations for swine, cattle, poultry, and salmon.

WP4, Economics and welfare, estimates the investment in animals, describes the burden of animal health and welfare problems as a health loss envelope, attributes and prioritizes the health loss envelope by diseases and health and welfare issues to target interventions and supports the economic assessment of the interventions at all levels. WP4 initiates a process that is health and welfare-focused, leading to information on the relative importance of disease and health and welfare problems and indicating where there is weak resource allocation ( Rushton *et al.*, 2018). The health loss envelope is based on developing input-output relationships for the current levels of production ( Rushton, 2008) and estimating a utopian level of production based on what is achievable within the current systems of genetics of the animals, feed available and management, minus the impacts caused by health and welfare problems. The differences between the current and utopian input-output relationship represents the net loss in production and the expenditure on animal health and welfare – the health loss envelope. The types of modelling approaches to achieve this have been explored for cattle and bovine respiratory disease ( Delabouglise *et al.*, 2017); for broiler chickens and coccidiosis ( Gilbert *et al.*, 2020) and for PRRS in pigs ( Thomann *et al.*, 2020). The information generated supports the selection of interventions (WP3) through careful cost-benefit analysis based on marginal cost and benefit calculations ( Gilbert *et al.*, 2020; Thomann *et al.*, 2020) that are informed by risk and uncertainty ( Rushton, 2008).

In WP5, Stakeholders, we explore how key and secondary actors, namely farm-staff and producers, veterinary and production advisors, engage with different data and information about diseases and what kind of decisions they make based on the data that is available to them. WP5 is made up of two sequential parts, ‘Exploration’ and ‘Experimentation and Evaluation’, based on the emerging concept of “Living Labs” for the co-creation and adoption of new services and technologies in multi-contextual real-life environments ( Bergvall-Kåreborn & Ståhlbröst, 2009). This framework has been successfully employed in a wide range of sectors (Healthcare, Transport, among others) for technology-led decision-support innovations. The key parts of the methodological approach of WP5 are the involvement of multiple actors in real-life settings and use of multi-method approaches informed from wider social science disciplines ( Klerkx *et al.*, 2019; Morgan *et al.*, 2001).

## Discussion

Endemic contagious diseases are the main worry in day-to-day animal health management for farmers and their veterinarians. The so-called production diseases are often caused by multiple pathogens and pose the largest threat to animal health and welfare and, thus, to the productivity of both terrestrial and aquatic species. For DECIDE, we focus on the three most important meat-producing terrestrial species (swine, poultry and cattle) and the most important aquatic species, salmon and trout in Europe. For young, growing animals, gastro-intestinal and respiratory tract infections are the most prevalent syndromes that cause much growth reduction, mortality, and use of medicine. In salmonids, respiration is impaired by gill diseases, and diseases causing circulatory failure and inflammation of gastrointestinal organs lead to growth reduction and mortality. The four species have different levels of integration of farming and thus of available data and differ in the level of data (animal, herd, unit, cage). However, the concept that a contagious disease spreads in an epidemiological unit and causes disease is the same. When syndromes are noted, stakeholders such as farmers, veterinarians or other animal health managers need to decide on actions to prevent further spread, diagnose, treat, vaccinate, or take preventive actions for the next round of production. These decisions and choices are not necessarily rational and often not based on a sound quantitative basis ( Kaler & Ruston, 2019). Why, how and when stakeholders decide to take action (the drivers) and why they do not (barriers) are some of the key-elements of the DECIDE project as depicted in the box “Stakeholder needs” in Figure 1. The social scientists in DECIDE ensure that in co-creation with the stakeholders, the project delivers suitable tools.

The DECIDE project leads to use of existing data for disease monitoring, to rank and prioritize disease control and decision-support for the stakeholders. DECIDE increases stakeholder awareness about infectious endemic disease control and identifies gaps for further training. The methods and framework developed in DECIDE are specific enough to support stakeholders for the focus species and syndromes in DECIDE, but can also be generalized across species, countries or diseases. Research gaps are identified and shared. The use of the co-created decision-support tools leads to improved decisions regarding disease control and thus results in improved sustainability, animal health and welfare. More efficient use of resources and less use of medicines will also positively impact human health and the environment.

## Data Availability

No data are associated with this article.
