# Supplementary material for: The DECIDE project: from monitoring and surveillance data to decision-support for farmers and veterinarians
Source: Open Res Eur. 2025 Aug 26;3:82. Originally published 2023 May 17. [Version 2] doi: 10.12688/openreseurope.15988.2 (PMC11109551; doi:10.12688/openreseurope.15988.2)
Supplement: Peer review history

# Reviewer report: Reviewer response for version 2

**DOI:** 10.21956/openreseurope.19167.r59138

**Published:** 2025-09-08

**Authors:** Philip A. Robinson

**Affiliations:** 1 Harper Adams University campus, Harper & Keele Veterinary School, Newport, Shropshire, UK

I am content that the points I raised have been addressed and the article can be approved.

Where applicable, are recommendations and next steps explained clearly for others to follow? (Please consider whether others in the research community would be able to implement guidelines or recommendations and/or constructively engage in the debate)

Partly

Does the article adequately reference differing views and opinions?

Partly

Are all factual statements correct, and are statements and arguments made adequately supported by citations?

Yes

Is the rationale for the Open Letter provided in sufficient detail? (Please consider whether existing challenges in the field are outlined clearly and whether the purpose of the letter is explained)

Yes

Is the Open Letter written in accessible language? (Please consider whether all subject-specific terms, concepts and abbreviations are explained)

Yes

Reviewer Expertise:

NA

I confirm that I have read this submission and believe that I have an appropriate level of expertise to confirm that it is of an acceptable scientific standard.

# Reviewer report: Reviewer response for version 1

**DOI:** 10.21956/openreseurope.17264.r39463

**Published:** 2024-05-07

**Authors:** Dirk Pfeiffer

**Affiliations:** 1 Centre for Applied One Health Research and Policy Advice, College of Veterinary Medicine and Life Sciences, City University of Hong Kong, Hong Kong, Hong Kong

DECIDE will produce decision support tools for early detection and control of selected diseases/syndromes in pigs, poultry and cattle as well as salmon and trout. It will integrate a variety of data sources and quantitative models to produce outputs that will be tailored to different stakeholders, also taking into account economic considerations.

 It is interesting that there is no mention of the environmental impact of production, disease and its control. DECIDE only seeks for optimisation of animal health and welfare, as well as monetary profit.  Admittedly, taking into account environmental impact would add complexity, but it should either be attempted or be mentioned as an issue in the discussion. 'Sustainability' and 'environment' are briefly mentioned in the last paragraph in the discussion. It may also be useful to refer to the widely accepted need for food system transformation and how DECIDE can, may be, support that. 

 In terms of analytical methodology, it would seem timely to consider the use of artificial intelligence approaches.

 The inclusion of social scientists in the project is to be commended, but I am unsure how an integration of the different types of knowledge or scientific evidence will be achieved.

Where applicable, are recommendations and next steps explained clearly for others to follow? (Please consider whether others in the research community would be able to implement guidelines or recommendations and/or constructively engage in the debate)

Yes

Does the article adequately reference differing views and opinions?

Yes

Are all factual statements correct, and are statements and arguments made adequately supported by citations?

Yes

Is the rationale for the Open Letter provided in sufficient detail? (Please consider whether existing challenges in the field are outlined clearly and whether the purpose of the letter is explained)

Yes

Is the Open Letter written in accessible language? (Please consider whether all subject-specific terms, concepts and abbreviations are explained)

Yes

Reviewer Expertise:

veterinary epidemiology

I confirm that I have read this submission and believe that I have an appropriate level of expertise to confirm that it is of an acceptable scientific standard.

# Reviewer report: Reviewer response for version 1

**DOI:** 10.21956/openreseurope.17264.r31756

**Published:** 2024-04-19

**Authors:** Philip A. Robinson

**Affiliations:** 1 Harper Adams University campus, Harper & Keele Veterinary School, Newport, Shropshire, UK

This article provides an interesting summary of the DECIDE project, a European research project designed to improve animal health and welfare by better utilizing a wide range of existing and novel on-farm data across pig, poultry, cattle and salmonid farms in Europe. The aim of the project is to improve decision-making around endemic contagious diseases of the respiratory and gastrointestinal systems of these species. 

 I think the title of the article should be slightly expanded to include the word 'monitoring' as the aim of the project is to utilize existing data, not just create new data i.e. 'The DECIDE project: from monitoring and surveillance data to decision support for farmers and veterinarians.' Surveillance is more active and intentional than monitoring, and I think both approaches to collecting and using data need to be considered. The word 'monitor' is indeed referred to at several points in the article.

 It would be helpful to see some more introduction to the topic in terms of previously published attempts to better utilize data on endemic disease perhaps elsewhere in the world, and to illustrate the challenges involved. This will also help to elucidate why this project is novel and pioneering.

 In terms of recommendations and next steps, the article is often written in the present tense, suggesting that the project is ongoing and has not yet reached its conclusion. This could be clarified for the benefit of the reader.

 There is no mention of WP6 and WP7 in the section 'Content of the work packages' - I think there should be some reference made to them here as they are shown in Fig. 2.

 Minor changes:

 1. Abstract: 'currently missing sufficient information on **the** prevalence and burden...'

 Introduction:

 2. Line 22: 'as well as **affecting** animal welfare'

 3. Lines 31-32: 'normal production process **es** indicative for infectious **disease** spread.'

 Page 7:

 4. Second paragraph, line 5: 'interventions and support **s** the economic assessment'

 5. Second paragraph, lines 21-23: remove the word 'by' before each of the sets of references (x3)

 6. Third paragraph, line 14: 'in real-life setting **s**'

 Discussion:

 7. 'For DECIDE, we focus on the three most important meat-producing terrestrial species...' - should the words 'in Europe' be added in here?

Where applicable, are recommendations and next steps explained clearly for others to follow? (Please consider whether others in the research community would be able to implement guidelines or recommendations and/or constructively engage in the debate)

Partly

Does the article adequately reference differing views and opinions?

Partly

Are all factual statements correct, and are statements and arguments made adequately supported by citations?

Yes

Is the rationale for the Open Letter provided in sufficient detail? (Please consider whether existing challenges in the field are outlined clearly and whether the purpose of the letter is explained)

Yes

Is the Open Letter written in accessible language? (Please consider whether all subject-specific terms, concepts and abbreviations are explained)

Yes

Reviewer Expertise:

Veterinary epidemiology; social science approaches to animal health and welfare

I confirm that I have read this submission and believe that I have an appropriate level of expertise to confirm that it is of an acceptable scientific standard, however I have significant reservations, as outlined above.

# Response

**Published:** 2024-05-03

**Authors:** Gerdien van Schaik

**Affiliations:** Utrecht University, Utrecht, Utrecht, The Netherlands

Dear Philip Robinson,

 Thank you for revising our paper and providing us with valuable comments for improvement of the paper. We agree that the data for the project is originating from both monitoring and surveillance activities. In the project we will use the data for decision support for disease management. We will change the title as the reviewer suggests to: 'The DECIDE project: from monitoring and surveillance data to decision support for farmers and veterinarians.' We included a few references in the Introduction to illustrate the challenges of using data for endemic disease control in animal production.  We hope that this additional text (in blue) is sufficient. The maximum length of the paper does not allow for much more examples. “Existing on-farm data are numerous and heterogeneous, greatly limiting their direct use by farms, while they could be used to monitor deviations from the normal production processes indicative for infectious disease spread. Baldin et al. (2021) conclude that the scientific community has not been successful in developing, disseminating, and promoting a sustained adoption of integrated decision support systems based on continuous, multiple data streams on dairy farms. While studies also show potential for data usage in disease surveillance and decision support systems (Hepworth et al., 2012; Niloofar et al., 2021). Also, sensor data or (video) imaging, are currently already available in specific farms and species ( Halachmi et al., 2019). For salmon production, imaging is already used quite extensively, while in terrestrial species, it is still in an early adoption phase. Salmon can thus serve as an example for the other species. Still, much progress could be made in managing endemic diseases by using available data in decision support systems useful to the farmers and their veterinarians (Kasab-Bachi et al., 2017; Rutten et al., 2013; Van Limbergen et al., 2020).”   We agree with the reviewer that it was not explicitly stated that the project is ongoing. We now mention in the introduction “The project started in 2021 and will reach conclusions in 2026”. We added: “The administrative workpackages WP6 and WP7 are for communication and dissemination (WP6) and overall project coordination (WP7).” All suggested minor changes were included in the revised version of the paper.

# Reviewer report: Reviewer response for version 1

**DOI:** 10.21956/openreseurope.17264.r38474

**Published:** 2024-04-08

**Authors:** Dennis Muhanguzi

**Affiliations:** 1 Makerere University, Kampala, Central Region, Uganda

I would like to congratulate the authors on a very lovely project proposal. I would only like to bring their attention to one point they might need to think about going forward;

 1. One of the production indices the authors propose to model in work package 4 is reduction in growth weight -see abstract; [in disease state] and probably the reverse in healthy state. Indices of productivity like weight gain are affected by many factors including genetics, nutrition and other management factors [environmental stressors]. Using such indices even when you are deploying robust modelling approaches might not tease out changes in body weight attributable to each of these collinear and / or interacting factors. It is therefore important that the authors either have inbuilt values assigned to each of the factors to explain their effects on the measured outcome [weight gain] or they contend with errors borne with use of such indices in modelling socio-economic impact of livestock diseases. Use of growth rate as a production index would require that the study design is longitudinal with data generated from "with disease" and "without disease" scenarios where herds are of similar genotypes and kept in somewhat homogeneous production systems. 

 A related point to the above is use of the phrase " possibly on antimicrobial use" in the second paragraph on page 5.  I would assume that Socio-economic impact assessments will consider the cost of treatments [including antibiotics] in high, medium and low disease burden herds / flocks / shoals. This being the case i would suggest this phrase should be changed to be in the affirmative.

Where applicable, are recommendations and next steps explained clearly for others to follow? (Please consider whether others in the research community would be able to implement guidelines or recommendations and/or constructively engage in the debate)

Yes

Does the article adequately reference differing views and opinions?

Yes

Are all factual statements correct, and are statements and arguments made adequately supported by citations?

Yes

Is the rationale for the Open Letter provided in sufficient detail? (Please consider whether existing challenges in the field are outlined clearly and whether the purpose of the letter is explained)

Yes

Is the Open Letter written in accessible language? (Please consider whether all subject-specific terms, concepts and abbreviations are explained)

Yes

Reviewer Expertise:

Molecular epidemiology | Veterinary Sciences

I confirm that I have read this submission and believe that I have an appropriate level of expertise to confirm that it is of an acceptable scientific standard.

# Response

**Published:** 2024-05-03

**Authors:** Gerdien van Schaik

**Affiliations:** Utrecht University, Utrecht, Utrecht, The Netherlands

Dear Dennis Muhanguzi,

 Thanks you for your thorough review of our paper. Please find our response to the issues you raise below. Ad 1. In summary, you are concerned about the use of growth rate as a production index. As you rightly mention it would require that the study design is longitudinal with data generated from "with disease" and "without disease" scenarios.  We will and are exploring options to look at the different issues and the points you raise with an adequate library of data on growth rates it should be possible to say something about an ideal growth rate under different environment and nutritional conditions and the type of genetics of the animal in order to understand what the gap is due to health. You will find that we are working on this as an approach within the Global Burden of Animal Diseases programme (Rushton et al, 2018, 2021, Gilbert et al, accepted).  We are also exploring a partial and disaggregated estimation of the burden of diseases through looking at the relative burden of the diseases and the potential reduction of the burden through control options. The main approaches we use are meta-analyses (Boeters et al., 2023) and stochastic modelling (Sorin-Dupont et al., 2023) based on parameters from literature. In some cases we also have retrospective empirical data in which we can determine the associations between the presence of syndromes (respiratory or gastro-intestinal), pathogens and, for example, (variation in) carcass weight or pleurisy condemnations at slaughter. When we mention that we consider the antimicrobial use (AMU) in the burden of animal diseases, we mean that we will not only consider the direct costs and benefits of AMU but also consider the unfavourable consequences of superfluous AMU for antimicrobial resistance. The costs of treatment (including antibiotics) are already included in the economic burden estimations. However, the indirect negative impact of AMU will also be indicated in decision support tools to inform decision makers. Since the tools are not yet in that stage of development, we propose to stick to ". possibly on antimicrobial use".
